# T172. MULTIMODAL QUANTIFICATION OF MEMORY CIRCUIT MICROSTRUCTURE IN FIRST EPISODE PSYCHOSIS

**DOI:** 10.1093/schbul/sby016.448

**Published:** 2018-04-01

**Authors:** Carolina Makowski, Christine Tardif, Gabriel Devenyi, Robert Amaral, Gabriella Buck, Ridha Joober, Ashok Malla, Jai Shah, Mallar Chakravarty, Martin Lepage

**Affiliations:** 1 McGill University; 2 McGill University, Douglas Mental Health University Institute; 3 Douglas Mental Health University Institute

## Abstract

**Background:**

Integrity of hippocampal subfield structure and associated limbic circuitry subserves various memory processes, a domain that is impaired in psychosis and an important predictor of functional outcome. We use a novel atlas that encapsulates both hippocampal subfields and surrounding white matter (WM), forming the ‘memory circuit’, to assess volumes with high-resolution MRI, and microstructure with quantitative T1 (qT1). Our aims were to examine 1) group by time interactions on memory measures and the memory circuit, and 2) explore the relationships between the chosen memory measures and limbic structures, informed by results from 1), in a longitudinal sample of first episode of psychosis (FEP) patients.

**Methods:**

Nineteen FEP and 20 controls with baseline and 3-month follow-up data were included. Logical Memory and Visual Reproduction Subscales of the Weschler Memory Scale, and MRI scans on a 3T scanner were collected. High-resolution T2-weighted images (0.64 mm3) were input to the MAGeT Brain algorithm to obtain volumes of hippocampal subfields and surrounding WM, defined by fimbria, alveus, fornix, and mammillary bodies. Mean qT1 values for each hippocampal subfield and WM structure were sampled from MP2RAGE (1 mm3) qT1 maps. Linear mixed models were used to assess group by time interactions on memory measures, volumes and qT1. To begin, total hippocampal volumes and WM structure for each hemisphere were examined using a Bonferroni correction for multiple comparisons, followed by post-hoc tests of individual subfields and WM structures. Linear models were then used to assess relationships between baseline memory and change in anatomical measures of interest in FEP. Models controlled for sex, education, age, and brain volume.

**Results:**

Significant group by time interactions emerged on bilateral mean WM qT1 (left: F1,65=9.3, p=.003; right: F1,65=10.6, p=.002), where it was found that within the FEP group, qT1 (relaxation time in ms) increased over the 3-month follow-up period. Looking at WM structures separately, the interaction was driven by qT1 changes in fimbria, fornix, and mammillary bodies bilaterally (p’s<.05). No significant group by time interactions were found with respect to volumes or memory, although a trend-like group by time interaction on right fornix volume was found (F1,64=5.6, p-uncorrected=.02). Finally, brain-behaviour relationships were explored, restricting our anatomical measure of interest to mean qT1 values within bilateral WM. Although no tests passed correction for multiple comparisons, there was a trend association between better delayed recall of Visual Reproduction and decreases in qT1 of combined WM on the right hemisphere (F1,11=3.72, p=.08), driven by changes in qT1 of the right fornix (F1,11=4.4, p=.06).

**Discussion:**

This study reveals significant microstructural changes in WM output circuitry of the hippocampus shortly after a FEP. Specifically, increases in qT1 were found within fimbria, fornix, and mammillary bodies bilaterally. Given that T1 relaxation times are typically shorter in WM, an increase in qT1 may reflect a combination of decreased myelin content and increased inflammation. Furthermore, preliminary data suggest better visual memory at baseline is associated with lower qT1 within WM microstructure over a 3-month period, suggesting that preserved non-verbal memory ability shortly after a FEP may manifest in a protective anatomical phenotype, particularly within the fornix. Given the importance of the hippocampal-fornix circuit in FEP, both with respect to memory and as a theorized hub of pathophysiology in psychosis, a better understanding of WM microstructure in relation to cognitive profiles in patients may offer a new perspective for treatment targets.

